# SE-Driven Dynamic Convolution for Adaptive EEG-Based Driver Fatigue Detection Across Spectral, Spatial, and Temporal Domains

**DOI:** 10.3390/s26092728

**Published:** 2026-04-28

**Authors:** Tianle Zhou, Jin Cheng, Jinbiao Zhang

**Affiliations:** 1 Mengxi Honors College, Jiangsu University, Zhenjiang 212013, China; 3240601040@stmail.ujs.edu.cn; 2 College of Mechanical and Electronic Engineering, Shandong Agricultural University, Tai’an 271018, China; 17866702267@163.com

**Keywords:** driver fatigue detection, EEG sensor signal processing, dynamic convolution, squeeze-and-excitation attention, lightweight neural network, cross-subject generalization, SEED-VIG

## Abstract

EEG-based driver fatigue detection faces three signal-level challenges: inter-subject spectral variability, coupled frequency–spatial–temporal dynamics that existing methods process independently, and dependence on a single labeling scheme. This paper presents DCAMNet, a lightweight CNN (12.3 K parameters) that addresses these challenges through three end-to-end blocks. An SE-driven dynamic convolution block adapts spectral sensitivity per sample via input-dependent kernel weighting—applied here for the first time to fatigue detection. A spatial convolution block encodes electrode-level cortical patterns, and a temporal attention block captures fatigue dynamics through windowed variance descriptors with group-wise attention scoring. DCAMNet was evaluated on SEED-VIG (PERCLOS labels) and MESD (reaction-time labels) under both subject-mixed and leave-one-subject-out (LOSO) protocols. Under LOSO cross-validation—the operationally relevant test that eliminates within-subject information leakage and simulates deployment on unseen drivers—DCAMNet achieved 85.43% accuracy on SEED-VIG with a 2.86-point advantage over the strongest baseline, and 79±5% accuracy on MESD with a 3-point advantage. As upper-bound estimates under the subject-mixed protocol, accuracy reached 97.47% (SEED-VIG) and 96.52% (MESD). With 1.35 ms inference latency on a standard GPU, the compact architecture suggests potential suitability for real-time embedded deployment, although on-device validation on representative automotive hardware remains necessary.

## 1. Introduction

Driver fatigue is a leading cause of serious traffic accidents worldwide; epidemiological estimates attribute 20% to 40% of crash events to fatigue [1]. Prolonged driving degrades alertness, reaction speed, and decision-making, raising the probability of vehicle control errors before any behavioral warning sign becomes visible [2]. A variety of sensing modalities have therefore been explored for continuous driver state monitoring. Approaches that rely on vehicle dynamics, steering behavior, or facial video are sensitive to environmental conditions—lighting variation, occlusion, road geometry—and cannot directly measure the neural state of the driver [3]. Among physiological modalities, electroencephalogram (EEG) signals stand out because they reflect cortical activity at millisecond-level temporal resolution and are less affected by external environmental factors [4]. As a sensing modality, however, scalp-recorded EEG is inherently noisy, non-stationary, and subject-dependent, so that robust sensor-driven fatigue detection requires signal-processing methods capable of adapting to inter-individual spectral variability while jointly exploiting the frequency, spatial, and temporal structure of the acquired waveforms.

As a continuous physiological sensor, EEG is well suited to fatigue monitoring for two reasons: it captures cortical state changes that precede overt behavioral degradation non-invasively, and its high temporal resolution permits the detection of transient fatigue-related fluctuations [5]. At the spectral level, fatigue-related EEG changes are well characterized—theta-band power (4–8 Hz) over frontal regions tends to increase, alpha-band power (8–13 Hz) shows a characteristic rise followed by suppression, and beta-band activity (13–30 Hz) decreases as the driver transitions from an alert to a fatigued state [4,6]. The precise frequencies at which these changes occur, their spatial distribution across electrodes, and their temporal dynamics, however, differ substantially across individuals owing to differences in neuroanatomy, sleep history, cognitive style, and habitual driving patterns [7]. This inter-subject variability in fatigue-related spectral patterns means that a feature representation calibrated for one individual may generalize poorly to another, constituting the primary signal-level challenge for any EEG-based fatigue detector that must operate without per-subject recalibration.

Early EEG-based fatigue detection systems relied on hand-crafted features derived from frequency-domain analysis [8]. Power spectral density (PSD) measures of theta and alpha bands were widely used to track fatigue-related band-power shifts, but PSD assumes signal stationarity and is sensitive to the non-stationary dynamics typical of real driving EEG [9]. Wavelet-based decompositions improved time–frequency resolution [10], and entropy measures such as multi-entropy fusion captured signal irregularity associated with fatigue [11,12]. Graph-based methods modeled inter-regional connectivity using complex network features and phase lag indices, revealing fatigue-related changes in functional brain organization [13,14]. Independent component analysis combined with entropy-based classifiers further improved subject-level discrimination [15]. Despite these advances, hand-crafted methods share two structural limitations: feature engineering is decoupled from the downstream classifier, preventing the pipeline from being optimized end-to-end for the detection task; and the fixed signal representations they produce cannot adapt to the individual differences in fatigue-related spectral patterns described above, which restricts generalization across subjects and recording conditions.

Deep learning has addressed the end-to-end limitation by learning feature representations directly from EEG data. CNNs have been applied to spatial and temporal EEG features for drowsiness classification [16], and spatio-temporal CNN designs have demonstrated the benefit of jointly modeling electrode and time dimensions [17]. Long short-term memory (LSTM) networks and compact interpretable CNNs have captured sequential dependencies in driver EEG under cross-subject settings [18], while Transformer-based architectures have modeled long-range temporal dependencies [19] and graph neural networks have encoded spatial relationships between electrodes through brain connectivity graphs [20,21]. Multi-scale and attention-augmented designs have further improved feature discriminability [22,23,24]. A recent comprehensive survey confirms that, despite the diversity of architectures proposed, three signal-level problems inherent in EEG sensor data remain insufficiently addressed [3]. First, virtually all CNN-based fatigue detectors use static convolution kernels that apply the same spectral filter weights to every input sample, regardless of the substantial inter-subject variability in fatigue-related spectral patterns; because EEG spectral signatures of fatigue differ across individuals, a fixed-kernel design cannot adapt to unseen drivers without per-subject recalibration [25]. Second, existing architectures typically handle frequency, spatial, and temporal features through independent branches or sequential modules without explicit cross-domain interaction, although fatigue manifests simultaneously across all three signal domains and the information lost at domain boundaries cannot be recovered by downstream fusion [26]. Third, most published methods are validated on a single dataset with one labeling scheme under subject-mixed protocols that permit within-subject information leakage, making it difficult to determine whether reported performance reflects genuine sensor-level correlates of fatigue or is inflated by subject-specific memorization and annotation artifacts [27,28]. Subject-independent evaluation under leave-one-subject-out protocols is therefore essential to establish the operational validity of any proposed method.

Squeeze-and-excitation (SE) networks and dynamic convolution provide effective mechanisms for input-dependent feature recalibration. In the EEG domain, SE-based feature fusion has improved motor imagery decoding by reweighting spectral and temporal channel responses according to input content [29,30]. Dynamic convolution—where multiple parallel kernels are aggregated with input-dependent weights instead of a single fixed kernel—has improved the cross-subject generalization of CNN-based EEG decoders, because the attention-weighted kernel mixture allows the model to select spectral filters appropriate for each individual sample [31,32]. These findings suggest that dynamic convolution is well suited to the inter-subject variability problem in EEG fatigue detection, yet this mechanism has not previously been applied to that task.

This paper presents DCAMNet, a lightweight CNN (12.3 K parameters on the SEED-VIG configuration) whose architecture is driven by the three signal-level challenges identified above rather than by model complexity. Each processing block targets a specific property of fatigue-related EEG sensor data: the dynamic convolution block addresses inter-subject spectral variability, the spatial convolution block captures region-specific cortical activation, and the temporal attention block models fatigue onset dynamics. The individual components—dynamic convolution, SE attention, spatial convolution, and depthwise separable temporal processing—draw on established techniques, but their design and parameterization are tailored to the signal-level properties of fatigue-related EEG: the dynamic convolution block operates on a nine-band spectral decomposition specific to fatigue-relevant frequency ranges (the nine sub-bands—δ, θ, $\alpha_{1}$, $\alpha_{2}$, $\beta_{1}$, $\beta_{2}$, $\gamma_{1}$, $\gamma_{2}$, and broadband—are defined in Section 2.2), and the temporal attention block uses variance-based descriptors matched to the non-stationary energy fluctuations characteristic of fatigue onset. To our knowledge, SE-driven dynamic convolution has not previously been applied to EEG-based fatigue detection. DCAMNet is evaluated on the MESD dataset, where fatigue is indexed by behavioral reaction time [33], and the SEED-VIG dataset, where labels are derived from PERCLOS-based eye tracking [34], to determine whether the learned representations generalize across labeling schemes. The main contributions are as follows:

- **Jointspectral–spatial–temporal signal representation.** DCAMNet integrates three sequential blocks into a single end-to-end pipeline that jointly processes the frequency, spatial, and temporal structure of fatigue-related EEG sensor data, avoiding the information loss incurred by independent-domain processing. Ablation experiments confirm that all three blocks provide non-redundant contributions (Section 3.4).
- **Adaptive spectral processing for inter-subject variability.** An SE-driven dynamic convolution block generates input-dependent kernel weights from a nine-band spectral decomposition aligned with fatigue-relevant frequency ranges, enabling per-sample spectral adaptation to accommodate the substantial inter-subject variability in fatigue-related EEG patterns. This mechanism has been validated for motor imagery decoding [31,32]; we adapt it to the fatigue detection setting with a domain-specific filter-bank design and SE branch operating on band-level energy descriptors.
- **Lightweight architecture with deployment potential.** With only 12.3 K trainable parameters and 1.35 ms inference latency on a standard GPU, DCAMNet is 2.5×–285× smaller than the evaluated baselines, indicating potential for real-time deployment on resource-constrained platforms subject to future on-device validation.
- **Evaluation under operationally relevant conditions.** DCAMNet is assessed on two benchmarks with different labeling schemes (MESD: reaction time; SEED-VIG: PERCLOS) under both subject-mixed and leave-one-subject-out (LOSO) protocols. The LOSO evaluation, which eliminates within-subject information leakage and simulates deployment on unseen drivers, is treated as the primary performance indicator. The MESD evaluation provides supplementary evidence that the learned representations generalize across labeling schemes.

Section 2 describes the datasets, preprocessing pipeline, and DCAMNet architecture with the signal-processing rationale behind each block. Section 3 reports the experimental results, including ablation studies and subject-independent evaluation. Section 4 discusses the findings, analyzes the learned representations, and addresses limitations. Section 5 draws conclusions.

## 2. Materials and Methods

### 2.1. Datasets

Two public benchmarks with different labeling schemes were used in this study: the Multimodal Eye State Detection (MESD) dataset, where fatigue labels are derived from behavioral reaction time, and the SEED-VIG dataset, where labels are derived from PERCLOS-based eye-tracking scores. Evaluating on both datasets makes it possible to assess whether the learned representations generalize across labeling schemes.

#### 2.1.1. Multimodal Eye State Detection (MESD) Dataset

The MESD dataset was collected by Cao et al. in 2015 and released publicly in 2019 [33]. It contains 62 recording sessions from 27 healthy subjects who performed a simulated driving task on a four-lane highway. During each 90 min session, the vehicle deviated laterally at random intervals and subjects steered it back on course. The elapsed time between deviation onset and the subject’s first corrective steering response was recorded as the reaction time, serving as a behavioral index of fatigue. EEG signals were acquired at 500 Hz from 30 channels.

Three-second EEG segments immediately preceding each deviation event were extracted and labeled with a combination of local reaction time (LRT) and global reaction time (GRT), defined in Equations (1) and (2), where i=1,2,… indexes successive deviation events within a session.

(1) LRT(i)=Act(i)−Dep(i)

(2) $$\mathrm{GRT}\left(i\right)=\mathrm{avg}\left\{\mathrm{LRT}(j)\;|\;j<i\;\mathrm{and}\;0\leq \mathrm{Dep}(i)-\mathrm{Dep}(j)\leq 90\;s\right\}$$

In Equation (1), Act(i) denotes the time at which the subject began responding to the *i*-th deviation, and Dep(i) denotes the onset time of that deviation. In Equation (2), GRT(i) is the mean LRT computed over all responses recorded in the 90-s window preceding the *i*-th deviation. Sessions in which fewer than two deviation events fell within that window were excluded from GRT computation, consistent with the original protocol [33]. The alert reaction time threshold ART was set to the 95th percentile of all LRT values, $\mathrm{ART}=P_{95}\;\left({\left\{\mathrm{LRT}\left(j\right)\right\}}_{j}\right)$, and segments were labeled according to Equation (3).

(3) $$\mathrm{Fatigue}\left(i\right)=\left\{\begin{array}{cc} 1 & \mathrm{if}\;\mathrm{LRT}(i)>2.5\;\mathrm{ART}\;\wedge \;\mathrm{GRT}(i)>2.5\;\mathrm{ART}\\ 0 & \mathrm{if}\;\mathrm{LRT}(i)<1.5\;\mathrm{ART}\;\wedge \;\mathrm{GRT}(i)<1.5\;\mathrm{ART} \end{array}\right.$$

Segments falling in the intermediate range [1.5ART,2.5ART] are ambiguous and were discarded following the original protocol [33]. Of the 27 subjects, 11 were retained after excluding sessions that did not yield at least 50 labeled segments per class under the criteria above. Subject indices follow the order of the original data file names. The resulting sample distribution is given in Table 1.

Dataset acquisition: https://doi.org/10.6084/m9.figshare.6427334.v5 (accessed on 23 January 2025).

#### 2.1.2. SEED-VIG Dataset

The SEED-VIG dataset was collected by Zheng and Lu at Shanghai Jiao Tong University [34]. Participants drove a simulated vehicle on a straight, monotonous four-lane highway displayed on an LCD screen—a route designed to induce fatigue through sustained low-demand driving. Data from 21 participants were retained (13 from noon sessions and 8 from evening sessions), with each session lasting approximately two hours. EEG and EOG signals were recorded with a Neuroscan SynAmps2 system (Compumedics Neuroscan, Charlotte, NC, USA) at 200 Hz from 17 EEG channels.

Eye tracking recorded fixation, saccadic movement, blinking, and eye-closure events. Let *s* index each successive 4 s epoch, and let $t_{\mathrm{blink}}\left(s\right)$, $t_{\mathrm{fixation}}\left(s\right)$, $t_{\mathrm{saccade}}\left(s\right)$, and $t_{\mathrm{closure}}\left(s\right)$ denote the total durations (in seconds) of blinking, fixation, saccadic movement, and full eye closure within epoch *s*, respectively. The total observable interval, which may be shorter than the 4 s epoch when eye-tracking data are temporarily unavailable, is defined in Equation (4):

(4) $$t_{\mathrm{interval}}\left(s\right)=t_{\mathrm{blink}}\left(s\right)+t_{\mathrm{fixation}}\left(s\right)+t_{\mathrm{saccade}}\left(s\right)+t_{\mathrm{closure}}\left(s\right)$$

PERCLOS (Percentage of Eyelid Closure) quantifies the proportion of each epoch during which the eyes were closed, as defined in Equation (5):

(5) $$\mathrm{PERCLOS}\left(s\right)=\frac{t_{\mathrm{closure}}\left(s\right)}{t_{\mathrm{interval}}\left(s\right)}$$

The EEG signals were segmented into 4 s epochs and assigned binary fatigue labels based on the PERCLOS threshold in Equation (6):

(6) $$\mathrm{Fatigue}\left(s\right)=\left\{\begin{array}{cc} 1 & \mathrm{if}\;\mathrm{PERCLOS}(s)>0.5\\ 0 & \mathrm{otherwise} \end{array}\right.$$

Dataset acquisition: https://bcmi.sjtu.edu.cn/home/seed/index.html (accessed on 23 January 2025).

### 2.2. EEG Preprocessing

Raw EEG signals from both datasets were preprocessed with the same pipeline before being fed to the model. A fourth-order zero-phase Butterworth bandpass filter (0.5–45 Hz) was applied to each channel to remove low-frequency drift and high-frequency noise while retaining the delta, theta, alpha, beta, and gamma bands implicated in fatigue [6,35]. No additional artifact rejection (e.g., ICA-based ocular or muscular artifact removal) was performed beyond the bandpass filter, consistent with the preprocessing reported in the original MESD and SEED-VIG publications [33,34]. This choice was made to preserve comparability with the benchmark protocols and to evaluate whether the proposed architecture can extract fatigue-relevant features from minimally processed EEG—a condition closer to practical online monitoring, where elaborate offline artifact rejection may not be feasible.

For the MESD dataset, filtered signals were segmented into non-overlapping 3 s epochs time-locked to the onset of each lateral deviation event (Section 2.1.1). For the SEED-VIG dataset, filtered signals were segmented into non-overlapping 4 s epochs aligned to the PERCLOS labeling intervals (Section 2.1.2). Each epoch, with dimensions C×T (*C*: number of EEG channels; *T*: number of time points), was treated as an independent sample and passed directly to the multi-view data representation module (Section 2.4).

A filter bank within the multi-view module further decomposed each preprocessed epoch into $N_{b}=9$ sub-bands: δ (0.5–4 Hz), θ (4–8 Hz), $\alpha_{1}$ (8–10 Hz), $\alpha_{2}$ (10–13 Hz), $\beta_{1}$ (13–20 Hz), $\beta_{2}$ (20–30 Hz), $\gamma_{1}$ (30–36 Hz), $\gamma_{2}$ (36–42 Hz), and broadband (0.5–45 Hz). This nine-band decomposition gives the dynamic convolution block simultaneous access to all fatigue-relevant spectral components at each time point. The design splits the alpha and beta bands into two sub-bands each because fatigue-related alpha enhancement and beta suppression exhibit distinct spectral profiles in their lower and upper sub-ranges [4,6]; the broadband channel provides a global energy reference that complements the band-specific information. The number of bands ($N_{b}=9$) balances spectral resolution against the dimensionality of the SE branch input: fewer bands would merge fatigue-relevant sub-band distinctions, while additional bands would increase the SE bottleneck dimension without adding physiologically distinct spectral content.

### 2.3. Overall Architecture

**Figure 1:**
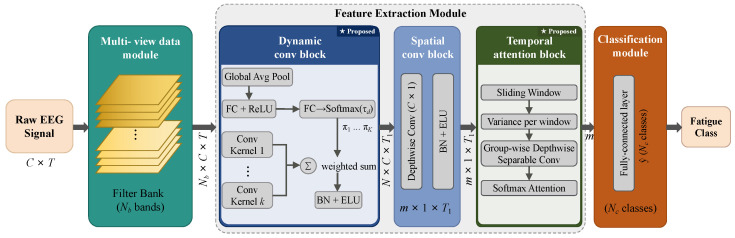
Overall architecture of DCAMNet. Blocks marked with ★ denote the proposed components.

DCAMNet comprises three modules: a multi-view data representation module, a feature extraction module, and a classification module. A bank of bandpass filters first decomposes the input EEG into multiple frequency-band representations, which the dynamic convolution block processes for spectral feature extraction. A spatial convolution block then integrates cross-channel information to encode region-specific cortical activation patterns. Finally, a temporal attention block segments the resulting time series into non-overlapping windows, models temporal dependencies through depthwise separable convolutions and group-wise self-attention, and feeds the aggregated features to a fully connected layer for binary classification. Figure 1 shows the overall architecture, and Table 2 lists the layer-wise parameter settings.

### 2.4. Multi-View Data Representation

Let *C* denote the number of EEG channels and *T* the number of time samples. The preprocessed EEG input $x\in R^{C\times T}$ is passed through a bank of $N_{b}$ bandpass filters $\left\{f_{1},f_{2},\ldots ,f_{N_{b}}\right\}$, yielding a multi-band tensor $x_{\mathrm{fb}}\in R^{N_{b}\times C\times T}$, where the subscript “fb” denotes the filter-bank representation and each slice along the first dimension corresponds to one frequency band. This tensor provides the downstream feature extraction module with band-specific spectral information relevant to fatigue detection.

### 2.5. Feature Extraction Module

The feature extraction module consists of three blocks—the dynamic convolution block, the spatial convolution block, and the temporal attention block—each operating on a different dimension of the EEG signal.

#### 2.5.1. Dynamic Convolution Block

**Figure 2:**
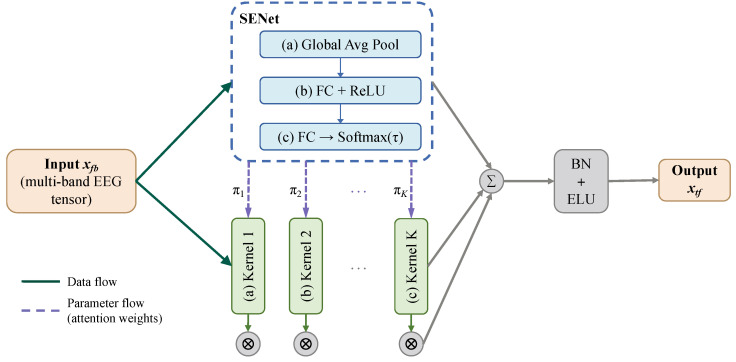
Structure of the dynamic convolution block. Solid grey arrows indicate data flow; dashed grey arrows indicate parameter flow (attention weights). The blue dashed frame highlights the SE branch (SENet) that generates the input-dependent kernel attention weights $\pi_{1},\ldots ,\pi_{K}$, and corresponds to one of the proposed components of DCAMNet (marked with ★ in Figure 1).

Because fatigue-related EEG spectral patterns vary substantially across individuals [7], a single fixed convolution kernel cannot capture the full range of inter-subject variability. The dynamic convolution block addresses this by aggregating *K* parallel kernels with input-dependent weights generated by an SE branch (Figure 2), enabling per-sample spectral adaptation.

Let $\left\{{\tilde{W}}_{1},{\tilde{W}}_{2},\ldots ,{\tilde{W}}_{K}\right\}$ denote the *K* parallel convolution kernels, where ${\tilde{W}}_{k}\in R^{N\times N_{b}\times 1\times L}$ is the weight tensor of the *k*-th kernel, *N* is the number of output feature maps, and *L* is the kernel length along the time dimension. The unit dimension in the third axis confines each kernel to operate along the time axis only, deferring spatial (channel-wise) mixing to the subsequent spatial convolution block. For a given input $x_{\mathrm{fb}}$, the kernels are combined with input-dependent weights:

(7) $$\tilde{W}\left(x_{\mathrm{fb}}\right)=\sum_{k=1}^{K}\pi_{k}\left(x_{\mathrm{fb}}\right)\;{\tilde{W}}_{k}$$

(8) $$\tilde{b}\left(x_{\mathrm{fb}}\right)=\sum_{k=1}^{K}\pi_{k}\left(x_{\mathrm{fb}}\right)\;{\tilde{b}}_{k}$$

The attention weights $\pi_{k}$ are produced by an SE branch. Global average pooling first compresses $x_{\mathrm{fb}}$ across the channel and time dimensions into a spectral descriptor $g\in R^{N_{b}}$:

(9) $$g_{b}=\frac{1}{CT}\sum_{c=1}^{C}\sum_{t=1}^{T}x_{\mathrm{fb}}\left(b,c,t\right),\;b=1,\;\ldots ,\;N_{b}$$

Two fully connected layers then map g to a *K*-dimensional logit vector: $z=W_{2}\;\mathrm{ReLU}\left(W_{1}g\right)$, where $W_{1}\in R^{\left(N_{b}/r\right)\times N_{b}}$ and $W_{2}\in R^{K\times (N_{b}/r)}$ are learnable weight matrices and *r* is the reduction ratio. The kernel attention weights are obtained via temperature-scaled Softmax with coefficient $\tau_{d}>0$ (set to 1.0 in all experiments):

(10) $$\pi_{k}\left(x_{\mathrm{fb}}\right)=\frac{\exp\;\left(z_{k}\left(x_{\mathrm{fb}}\right)/\tau_{d}\right)}{\sum_{j=1}^{K}\exp\;\left(z_{j}\left(x_{\mathrm{fb}}\right)/\tau_{d}\right)}$$

By construction, $\pi_{k}\left(x_{\mathrm{fb}}\right)\in \left[0,1\right]$ and $\sum_{k=1}^{K}\pi_{k}\left(x_{\mathrm{fb}}\right)=1$. A smaller $\tau_{d}$ produces a sparser weight distribution, concentrating the effective kernel on fewer basis kernels. After kernel aggregation, the output passes through batch normalization and ELU activation, yielding $x_{\mathrm{tf}}\in R^{N\times C\times T_{1}}$.

#### 2.5.2. Spatial Convolution Block

Because fatigue-related cortical activation tends to be spatially localized, particularly over central and prefrontal regions, the spatial convolution block applies *m* filters of size C×1 along the channel dimension of $x_{\mathrm{tf}}$. Each filter integrates information across all electrodes while preserving the time–frequency structure inherited from the dynamic convolution block. Batch normalization and ELU activation follow, producing *m* single-channel time series $x_{\mathrm{sc}}\in R^{m\times 1\times T_{1}}$.

#### 2.5.3. Temporal Attention Block

**Figure 3:**
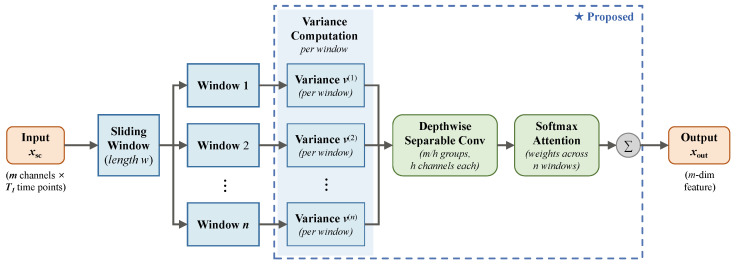
Structure of the temporal attention block. The ★ symbol indicates that this block is one of the components proposed in this work (consistent with the marking convention introduced in Figure 1). The blue dashed frame encloses the three sub-modules—per-window variance computation, depthwise separable convolution, and softmax attention—that together constitute the proposed temporal attention mechanism.

Fatigue-related EEG changes are non-stationary: they unfold over time with gradual onset and periodic fluctuations that vary in duration and intensity [4]. A global temporal descriptor would average out these local dynamics. To preserve them, the temporal attention block divides $x_{\mathrm{sc}}$ into $n=\lfloor T_{1}/w\rfloor$ non-overlapping windows of length *w* (Figure 3).

Within each window, the variance of each channel over the *w* time points serves as a local energy descriptor:

(11) $$v_{p}\left(i\right)=\frac{1}{w}\sum_{t=pw}^{(p+1)w-1}{\left(x_{\mathrm{sc}}\left(i,1,t\right)-\mu_{p}\left(i\right)\right)}^{2},\;p=0,\;1,\;\ldots ,\;n-1$$

where $\mu_{p}\left(i\right)=\frac{1}{w}\sum_{t=pw}^{(p+1)w-1}x_{\mathrm{sc}}\left(i,1,t\right)$ is the mean of channel *i* within window *p*, and $n=\lfloor T_{1}/w\rfloor$ is the total number of windows.

The variance descriptors $\left\{v_{0},v_{1},\ldots ,v_{n-1}\right\}$ ($v_{p}\in R^{m}$) are then processed by group-wise depthwise separable convolutions. The *m* channels are split into m/h groups of *h* channels each; within every group, a depthwise convolution operates independently across the *n* windows, and the raw outputs are normalized via Softmax to produce attention weights. The weighted outputs of all groups are concatenated into $x_{\mathrm{out}}\in R^{m}$:

(12) $$x_{\mathrm{out}}\left(c\right)=\sum_{q=0}^{n-1}U\;\left(c\;\mathrm{mod}\;h,\;q\right)\;v_{q}\left(c\right),\;c=0,\;1,\;\ldots ,\;m-1$$

where $U\in R^{h\times n}$ is the depthwise convolution weight matrix shared across all groups, U(j,q) denotes its (j,q)-th entry, and cmodh maps each output channel to its group-local index within a group of size *h*. The resulting vector $x_{\mathrm{out}}$ is passed to the fully connected layer for classification.

## 3. Results

DCAMNet was evaluated on both datasets using four metrics—accuracy, precision, recall, and F1 score. All experiments were conducted in Python 3.8 with PyTorch 1.12.

### 3.1. Implementation Details

All models were optimized with the Adam optimizer [36], using an initial learning rate of $1\times 10^{-3}$, $\beta_{1}=0.9$, $\beta_{2}=0.999$, and weight decay $1\times 10^{-4}$. The learning rate was halved every 50 epochs via a step decay schedule. Training ran for up to 300 epochs with a batch size of 64. Early stopping with a patience of 30 epochs was applied on validation loss, where 10% of the training data was held out for validation. Dropout at a rate of 0.5 was applied before the fully connected classification layer.

For DCAMNet, the dynamic convolution block used K=4 parallel kernels of length L=64, producing N=16 output feature maps, with SE reduction ratio r=4 and temperature $\tau_{d}=1.0$. The spatial convolution block used m=16 filters, and the temporal attention block used window length w=25 with h=4 group channels. The window length corresponds to 125 ms at the 200 Hz sampling rate of SEED-VIG, a duration consistent with the temporal scale of EEG microstate transitions and short-term energy fluctuations associated with fatigue onset [5]. The fully connected layer mapped the *m*-dimensional feature vector to $N_{c}=2$ output classes.

Five baseline architectures were implemented for controlled comparison. **CNN**: two temporal convolutional layers (64 and 128 filters, kernel size 5), each followed by batch normalization and ReLU, a global average pooling layer, and a fully connected classifier. **LSTM**: a two-layer bidirectional LSTM with 64 hidden units per direction, followed by a fully connected layer on the final hidden state. **Transformer**: a two-layer encoder with four attention heads, embedding dimension 64, and a classification token pooled through a linear head. **ResNet**: a one-dimensional ResNet-18 with residual blocks of [16, 32, 64, 128] filters, adapted from the standard image classification design to one-dimensional time-series input. **DBN**: a three-layer deep belief network with [256, 128, 64] hidden units, pretrained layer-wise with contrastive divergence and fine-tuned with backpropagation; because the DBN operates on the flattened input vector (C×T=13,600 dimensions on SEED-VIG), the first layer alone contributes approximately 3.48 M parameters, accounting for its high parameter count (this table is presented and discussed in Section 4.1). All baselines received the standard single-band preprocessed EEG input (1×C×T), following conventional practice for each architecture, and were trained with the identical optimizer, learning rate schedule, early stopping criterion, and data splits. DCAMNet received the nine-band filter-bank tensor ($N_{b}\times C\times T$) as described in Section 2.4; the multi-band decomposition is an integral part of the proposed method rather than a generic preprocessing step. Hyperparameters for each baseline were selected via validation-set performance from a predefined search grid; full architectural specifications and search ranges are provided in the Supplementary Material.

The total number of trainable parameters in DCAMNet was approximately 28.5 K on the MESD configuration (30 channels, 1500 time points) and 12.3 K on the SEED-VIG configuration (17 channels, 800 time points).

To ensure full reproducibility, all experiments used five fixed random seeds (0, 1, 2, 3, 4) applied consistently to PyTorch (version 1.12; PyTorch Foundation, San Francisco, CA, USA), NumPy (version 1.23; NumFOCUS, Austin, TX, USA), and Python’s (version 3.8; Python Software Foundation, Wilmington, DE, USA) random module at the beginning of each run. For subject-mixed experiments, stratified random sampling was performed using scikit-learn’s (version 1.1; scikit-learn developers, distributed via PyPI) train_test_split

### 3.2. Detection Results on the MESD Dataset

The MESD evaluation serves as supplementary evidence that DCAMNet’s learned representations generalize across labeling schemes; SEED-VIG (Section 3.3) is the primary benchmark. From the 2022 labeled segments across 11 subjects (Table 1), 400 fatigued and 400 alert segments were randomly sampled with equal representation from each subject to ensure class balance, yielding 800 samples. These were split 7:3 into training and test sets by stratified random sampling (560 training, 240 test). Because the split was performed at the segment level, segments from the same participant may appear in both sets. This subject-mixed protocol provides an upper-bound estimate; the subject-independent LOSO evaluation is reported at the end of this section.

DCAMNet achieved 96.52% accuracy, 95.23% precision, 95.87% recall, and 95.55% F1 score on MESD (Table 3), with all four metrics exceeding 95%. For baseline comparison, the five methods described above were trained and evaluated under identical conditions, each run five times (Table 4).

DCAMNet outperformed all baselines, achieving a mean accuracy of 96.41±0.47% versus the strongest baseline DBN (94.53±0.55%), a margin of 1.88 percentage points. The advantage on MESD was larger than on SEED-VIG, consistent with the shorter 3 s segments in MESD introducing greater inter-trial spectral variability—a condition under which per-sample kernel adaptation is expected to be most beneficial.

On the 240-sample MESD test set, DCAMNet produced seven false alarms (alert misclassified as fatigued) and only one missed detection (fatigued misclassified as alert), yielding an overall error rate of 3.33%. The low missed-detection count (1 out of 120 fatigued samples, 0.83%) is operationally favorable, as it indicates the model rarely fails to flag a genuinely fatigued driver under the reaction-time labeling scheme.

To assess subject-independent generalization on MESD, an 11-fold LOSO cross-validation was conducted under the same training protocol. Table 5 reports the results for all six methods.

DCAMNet achieved 79±5% LOSO accuracy on MESD, a 3-point advantage over the second-best method (DBN, 76±5%). The 17.52 percentage-point drop from the subject-mixed result (96.52% → 79%) is larger than the corresponding drop on SEED-VIG (11.92 points), reflecting the smaller number of training subjects available per fold (10 vs. 20) and the higher label noise inherent in reaction-time-based annotation. Subject 7, which contributed only 51 segment pairs, yielded the lowest fold accuracy (69%), consistent with the expectation that limited per-subject data amplifies cross-subject generalization difficulty. No prior work has reported LOSO results on MESD under the same LRT/GRT labeling criteria; the MESD LOSO evaluation therefore serves primarily as supplementary evidence that DCAMNet retains its cross-subject advantage under an independent labeling scheme.

### 3.3. Detection Results on the SEED-VIG Dataset

On SEED-VIG, 800 balanced samples (400 per class) were drawn by randomly selecting an equal number of fatigued and alert epochs from each of the 21 participants. An 8:2 stratified split at the epoch level produced 640 training and 160 test samples. As on MESD, this is a subject-mixed protocol; subject-independent generalization is evaluated separately in Section 3.5.

DCAMNet achieved 97.47% accuracy, 96.71% precision, 95.28% recall, and 95.99% F1 score (Table 6). The near-equal precision and F1 score indicate that false alarms and missed detections were kept at comparable levels. Because segments from the same participant may appear in both training and test sets, these figures represent an upper bound; Section 3.5 provides a more conservative, subject-independent estimate.

To verify that the subject-mixed results are not sensitive to the specific random subset selection, a re-sampling stability experiment was conducted on both datasets. Five independent balanced subsets (400 fatigued + 400 alert) were drawn using different random seeds (100, 200, 300, 400, 500), each split and trained under the same protocol as the main experiment. Table 7 reports the results.

On SEED-VIG, the mean accuracy over five independent re-samplings was 97.29±0.25%, closely matching the original result (97.35±0.42%). On MESD, the re-sampling mean was 96.34±0.31%, again consistent with the original (96.41±0.47%). The low re-sampling standard deviations (≤0.31%) confirm that the subject-mixed performance is stable across different random subset selections and that the conclusions do not depend on a particular sampling realization. Note that the LOSO evaluation (Section 3.5), which uses all eligible data from each subject without subsampling, remains the primary performance indicator.

### 3.4. Ablation Study

To quantify the contribution of each module, ablation experiments were conducted on SEED-VIG under the same 8:2 stratified split, with each variant run five times using different random seeds.

#### 3.4.1. Module-Level Ablation

Four variants were compared with the full DCAMNet: (i) replacing the SE-driven dynamic convolution with a single static kernel (K=1, no SE branch); (ii) removing the spatial convolution block; (iii) removing the temporal attention block and replacing it with global average pooling; and (iv) a minimal baseline using only the static convolution and a fully connected classifier. Results are shown in Table 8.

Comparing the full model with variant (i) isolates the effect of dynamic convolution: the 2.22 percentage-point accuracy gain confirms that input-dependent kernel aggregation captures inter-sample spectral variability that a fixed kernel cannot represent. Removing the spatial block (variant (ii)) reduced accuracy by 1.47 percentage points, and removing the temporal attention block (variant (iii)) by 1.25 percentage points, indicating that cross-channel cortical patterns and within-segment temporal dynamics each carry discriminative information that the other two domains cannot recover. The minimal baseline (variant (iv)) scored 4.70 percentage points below the full model—substantially more than the gap from variant (i) alone (2.22 percentage points). This pattern indicates that the three blocks interact complementarily: dynamic convolution provides per-sample spectral adaptation, but its benefit is amplified when combined with spatial encoding and temporal attention. The result also addresses the question of whether simply inserting a dynamic convolution module into an existing CNN would yield comparable performance; the ablation evidence indicates it would not.

#### 3.4.2. Hyperparameter Sensitivity

The number of parallel kernels *K* and the SE reduction ratio *r* are the two key hyperparameters of the dynamic convolution block. Their effects were evaluated on SEED-VIG under the same protocol (Table 9 and Table 10).

Accuracy increased monotonically from K=2 to K=4, peaking at 97.35%. At K=5, accuracy decreased slightly, suggesting that four kernels provide sufficient spectral diversity without introducing redundant parameters.

The optimal ratio was r=4. A smaller ratio (r=2) increases SE branch capacity but risks overfitting on the small training set, while a larger ratio (r=8) over-compresses the spectral descriptor.

#### 3.4.3. Frontal Channel Ablation

Because SEED-VIG labels are derived from PERCLOS (eye closure proportion) and no artifact rejection beyond bandpass filtering was applied, part of the model’s performance could, in principle, reflect ocular artifact contamination rather than cortical fatigue information. To assess the extent of this potential confound, DCAMNet was re-evaluated after removing the channels most susceptible to electrooculographic (EOG) leakage, without retraining the remaining architecture (Table 11).

Removing the two frontopolar channels (FP1 and FP2), which are most directly affected by eye-movement artifacts, reduced subject-mixed accuracy by only 1.03 percentage points and LOSO accuracy by 2.42 percentage points. This modest degradation indicates that the model does not critically depend on EOG-contaminated signals for its predictions. Removing all seven frontal channels produced a larger drop (5.31 points subject-mixed; 7.07 points LOSO), which is expected because frontal regions—particularly prefrontal cortex—are established generators of fatigue-related theta enhancement [4,6]; the loss therefore reflects the removal of genuine cortical information, not merely the elimination of ocular artifacts. This interpretation is consistent with the frequency band importance analysis reported later in Section 4.2.4, where the theta band (4–8 Hz) provided the highest single-band accuracy (92.33%), while the delta band (0.5–4 Hz)—the range most overlapping with EOG spectral content—ranked only fifth. These observations are suggestive but do not by themselves constitute definitive proof that ocular artifacts play no role; a conclusive separation would require dedicated artifact rejection as discussed in Section 4.3.

### 3.5. Subject-Independent Evaluation

The subject-mixed splits used in Section 3.2 and Section 3.3 allow segments from the same participant to appear in both training and test sets, which can inflate accuracy through within-subject pattern similarity. In any real-world deployment of an EEG-based fatigue sensor, the system must generalize to drivers whose data were not available during training; subject-independent evaluation therefore constitutes the operationally relevant test. All six methods (including LSTM and Transformer) were evaluated under this protocol, with the SEED-VIG LOSO results summarized in Table 12; unlike the subject-mixed setting, several folds contain fewer than 30 samples per class for the held-out subject, which disproportionately affects models with higher data requirements.

All methods showed a substantial accuracy drop relative to the subject-mixed setting, confirming the well-documented difficulty of cross-subject generalization in EEG-based fatigue detection. DCAMNet achieved 85.43±6.51% (95% CI: [82.63, 88.23])—11.92 percentage points below its subject-mixed result, but 2.86 percentage points above the strongest baseline (DBN, 82.57±7.23%). A fold-level paired *t*-test confirmed that DCAMNet’s advantage over DBN was statistically significant (t=11.27, $p=4.6\times 10^{-10}$), and a Wilcoxon signed-rank test yielded a consistent conclusion (W=231, $p=1.2\times 10^{-5}$). The effect size was large (Cohen’s d=2.46), indicating that the improvement is not attributable to random fold variation. The magnitude of the effect size reflects the consistent per-fold performance gap observed across most of the 21 subjects, rather than a few outlier folds driving the aggregate statistic. That the advantage is retained under LOSO supports the claim that dynamic convolution provides meaningful adaptation to inter-subject spectral variability. The fold-level standard deviation of ±6.51% reflects the substantial individual differences in fatigue-related EEG patterns reported in the neurophysiological literature [7], and the absolute accuracy level confirms that subject-independent fatigue detection remains an open challenge for all methods.

To provide a more detailed view of classification performance under the LOSO protocol, Table 13 reports the aggregate confusion matrix obtained by summing the per-fold predictions across all 21 held-out subjects.

The aggregate sensitivity (recall for the fatigued class) was 890/(890+160)=84.8%, and the aggregate specificity (recall for the alert class) was 900/(900+150)=85.7%. The near-symmetric sensitivity–specificity balance indicates that the model does not systematically favor one class over the other at the population level, although per-subject variation exists (see the per-subject analysis in Section 4.2.1).

Nevertheless, the 85.43% LOSO accuracy with a 12.3 K-parameter model suggests that lightweight adaptive signal processing can provide a meaningful degree of cross-subject generalization without requiring subject-specific calibration data—a desirable property for prospective deployment of EEG-based fatigue sensors.

## 4. Discussion

### 4.1. Comparison with Other Methods

Beyond absolute classification accuracy, the primary aim of the comparative evaluation is to determine whether adaptive spectral processing improves robustness to inter-subject EEG variability while maintaining a model footprint compatible with sensor-oriented deployment. DCAMNet was compared against five baselines on SEED-VIG under identical training conditions, with each model run five times using different random seeds (Table 14).

Across five runs, DCAMNet consistently achieved the highest mean accuracy (97.35±0.42%) and F1 score (95.87±0.48%). Under the subject-mixed protocol, DCAMNet led on accuracy, precision, and F1 score across both datasets and all five runs, a pattern unlikely to arise from random seed variation alone. More importantly, fold-level paired statistical tests on the LOSO evaluation (21 folds) confirmed a significant advantage over DBN (paired *t*-test: t=11.27, $p=4.6\times 10^{-10}$; Wilcoxon signed-rank: W=231, $p=1.2\times 10^{-5}$; Cohen’s d=2.46), providing strong evidence that the improvement generalizes across subjects rather than reflecting random variation.

Relative to DBN, the 0.61 percentage-point accuracy gain corresponds to an approximate 19% reduction in the mean misclassification rate. On precision, DCAMNet (96.58%) exceeded ResNet (95.67%) by 0.91 percentage points, indicating fewer false fatigue alarms. DBN achieved slightly higher mean recall (95.65% vs. 95.17%), consistent with a generative model’s tendency to favor sensitivity; DCAMNet led on accuracy, precision, and F1 score, making it the more balanced detector in settings where false alarms carry operational cost.

The five baselines were chosen to represent distinct learning paradigms—spatial convolution (CNN, ResNet), sequential modeling (LSTM), long-range self-attention (Transformer), and generative pretraining (DBN)—rather than to replicate specific published fatigue detectors. This paradigm-level comparison isolates the architectural contributions of DCAMNet from confounds that would arise when reimplementing published domain methods whose source code is not publicly available: undocumented hyperparameter choices, preprocessing details, and sample-selection criteria could inadvertently bias the comparison in either direction. The controlled results in Table 14 therefore prioritize reproducibility, while Table 15 provides cross-method context from published figures.

Under the stratified-split protocol, DCAMNet (97.47%) is numerically higher than the previously reported MATCN-GT result (93.67%) [40] by 3.80 percentage points; however, because the two methods differ in preprocessing, sample selection, and input representation, this gap should not be interpreted as a controlled comparison. Under LOSO, DCAMNet (85.43%) is comparable to PLI-GAT (85.53%) [14], confirming that cross-subject generalization remains a shared challenge across methods.

The numerical difference between DCAMNet and two recent methods—SFT-Net [24] and MSDA-Net [28]—may be partly attributable to differences in input representation, although a definitive comparison would require matched experimental conditions. SFT-Net constructs a 4D tensor from differential entropy (DE) computed over five standard bands before learning begins, fixing the spectral decomposition at preprocessing time. DCAMNet instead applies SE-driven dynamic convolution directly to the multi-band filter-bank output, allowing per-sample adjustment of spectral weights. The ablation study (Table 8) shows that dynamic convolution alone accounts for 2.22 of the 4.70 percentage-point advantage over the static-only baseline, with the spatial and temporal blocks contributing the remainder—indicating that the performance gain arises from the joint architecture rather than from any single component. MSDA-Net projects DE features onto a 2D topographic map, requiring the electrode layout to be fixed at design time; DCAMNet’s spatial convolution block operates directly on the channel dimension without 2D remapping, making it agnostic to electrode geometry and enabling application across datasets with different montages.

Table 16 compares computational cost across all methods on the SEED-VIG configuration.

DCAMNet has the fewest parameters (12.3 K) among all methods—2.5× fewer than ResNet, 4.4× fewer than CNN and LSTM, and 5.5× fewer than Transformer. Its FLOPs (18.6 M) are higher than DBN (7.3 M) but lower than all convolutional and recurrent baselines (24.2–115.6 M). The comparison is not strictly input-equivalent because DCAMNet receives the nine-band filter-bank tensor while baselines operate on the single-channel signal; the richer input enables the spectral processing that underlies the accuracy advantage but also increases per-sample computation. LSTM incurs the highest latency (4.86 ms) due to sequential processing, whereas DCAMNet’s fully convolutional architecture permits parallel execution at 1.35 ms per sample—three orders of magnitude below the 3–4 s epoch durations used in both datasets, confirming that the dynamic convolution mechanism does not preclude near-real-time operation.

The compact footprint stems from three design choices: the dynamic convolution block operates on the band dimension ($N_{b}=9$) rather than the full time series; the spatial block uses C×1 filters instead of dense cross-channel matrices; and the temporal attention block processes variance descriptors over non-overlapping windows rather than attending over all $T_{1}$ time steps. From a deployment perspective, the 12.3 K parameter count and sub-2 ms GPU latency are suggestive of compatibility with automotive-grade embedded processors [41]; however, GPU inference times do not directly predict performance on microcontroller or FPGA targets, and on-device profiling under real hardware memory, power, and latency constraints remains a necessary validation step before any deployment claim can be made.

A notable finding from the MESD LOSO evaluation (Table 5) is the reversal of baseline rankings relative to the subject-mixed setting: DBN, the strongest baseline under subject-mixed conditions, dropped to 76±5% under LOSO—below ResNet and Transformer. This reversal can be attributed to three factors. First, DBN contains approximately 3.5 M parameters, making it highly prone to overfitting when each LOSO fold provides only 10 training subjects. Second, unlike convolutional models that preserve spatial and temporal structure, DBN operates on flattened EEG vectors (C×T=13,600 dimensions), discarding structural priors that aid cross-subject generalization. Third, the MESD dataset contains fewer subjects (11 vs. 21 in SEED-VIG) and exhibits higher label noise from reaction-time-based annotation, amplifying the generalization difficulty for high-capacity models. By contrast, DCAMNet’s compact architecture (12.3 K parameters on SEED-VIG, 28.5 K on MESD) and inductive biases—spectral adaptation, spatial encoding, temporal attention—mitigate overfitting and preserve the structural information needed for cross-subject transfer.

### 4.2. Analysis of Learned Representations

#### 4.2.1. Per-Subject Performance (LOSO)

To assess how uniformly the model generalizes across individuals, per-subject accuracy was computed from the LOSO evaluation on SEED-VIG, where each value corresponds to the fold in which that subject served as the held-out test set (Table 17).

Fourteen of the 21 subjects exceeded 83% accuracy, and six exceeded 88%, indicating that the model generalizes well to the majority of individuals. Three subjects fell below 80%, and closer inspection of these cases reveals distinct sources of difficulty.

Subject 15 yielded the lowest fold-level accuracy (73.26%). This participant’s PERCLOS distribution was concentrated near the classification threshold of 0.5, with a high proportion of transitional-state epochs where the boundary between fatigued and alert states is inherently ambiguous. Under such near-threshold labeling, even a perfect cortical-state decoder would face irreducible classification noise.

Subject 6 (78.45%) was recorded in an evening session, a condition associated with higher baseline drowsiness and reduced spectral contrast between alert and fatigued states. The compressed dynamic range of fatigue-related spectral changes in evening recordings may reduce the discriminability available to the dynamic convolution block.

Subject 12 (79.33%) contributed a relatively small number of labeled epochs, limiting the model’s exposure to that individual’s EEG patterns during the other 20 training folds. More broadly, the three low-accuracy subjects share the characteristic that their fatigue-related EEG patterns deviate substantially from the population-level representation learned during training—a known limitation of subject-independent EEG decoding [7]. These cases suggest that subject-adaptive calibration or domain adaptation techniques may be necessary to close the remaining cross-subject gap.

#### 4.2.2. Error Analysis

Under the subject-mixed protocol on SEED-VIG (160 test samples, five runs aggregated via majority voting), DCAMNet produced two false alarms and four missed detections (error rate 3.75%). The asymmetry—more missed detections than false alarms—warrants attention from a safety perspective, as failing to flag a genuinely fatigued driver carries more severe consequences than a false alarm. Under the LOSO protocol the missed-detection rate increased for the low-accuracy subjects identified in Table 17 (Subjects 6, 12, and 15), indicating that these individuals’ fatigue-related EEG patterns are not well captured by the population-level model. Adjusting the classification threshold toward higher recall may therefore be preferable in deployment settings where missed detections carry greater operational risk.

#### 4.2.3. Dynamic Convolution Kernel Weight Distribution

To verify that the dynamic convolution block produces input-dependent kernel weights, the attention coefficients $\left\{\pi_{1},\pi_{2},\pi_{3},\pi_{4}\right\}$ were recorded for all test samples and grouped by class. For alert samples, the mean distribution was [0.31,0.27,0.24,0.18], indicating relatively balanced use of all four kernels. For fatigued samples, the distribution shifted to [0.18,0.22,0.28,0.32], with greater weight on kernels 3 and 4. This class-dependent shift indicates that the SE branch learned to route alert and fatigued samples through different kernel combinations, consistent with the known spectral differences between these states: fatigued EEG is characterized by elevated theta and alpha power, which specific kernel parameterizations may capture more effectively.

#### 4.2.4. Frequency Band Importance

To assess the contribution of individual bands, a single-band masking evaluation was conducted on the SEED-VIG test set: in each condition, only one filter-bank output was retained while the remaining eight were zeroed, and the masked tensor was passed through the trained network without retraining (Table 18).

Theta (θ, 4–8 Hz) achieved the highest single-band accuracy (92.33%), followed by broadband (94.00%) and $\alpha_{1}$ (91.06%). The prominence of theta is consistent with established findings that frontal theta power increases as cognitive vigilance declines [4,6]. The alpha sub-bands ranked third and fourth, reflecting the alpha enhancement associated with reduced alertness [42].

The gamma sub-bands yielded the lowest accuracies (84.83% and 83.48%), contrasting with methods such as SFT-Net [24] that report gamma DE as the most discriminative feature. The difference is attributable to the input representation: DE amplifies energy contrast in high-frequency bands, whereas DCAMNet processes the filtered time series directly, where gamma signals have a lower signal-to-noise ratio in driving EEG. The delta band (89.37%) outperformed both beta sub-bands, possibly reflecting intermittent delta activity associated with microsleep episodes during monotonous driving [4].

The full model (97.47%) exceeded the best single-band condition (broadband, 94.00%) by 3.47 percentage points, confirming that the nine-band decomposition provides complementary spectral information that no individual band can replicate. This result validates both the filter-bank design (Section 2.2) and the dynamic convolution block’s role in learning to weight bands adaptively per sample.

### 4.3. Limitations and Future Work

**Baseline selection.** The controlled comparison uses general-purpose architectures rather than reimplementations of domain-specific fatigue detectors such as MATCN-GT or SFT-Net. This choice was made because none of the recent domain methods in Table 15 provide open-source code; reimplementing complex architectures from textual descriptions alone risks introducing unintended differences that could bias the comparison. Future work should include domain-specific baselines as their implementations become publicly available.

**On-device validation.** DCAMNet’s inference time of 1.35 ms on an RTX 3090 GPU and its 12.3 K parameter footprint are promising for embedded deployment, but on-device validation on representative automotive-grade hardware (e.g., ARM Cortex-M microcontrollers or FPGA platforms) has not yet been performed. Model compression (pruning, quantization, knowledge distillation) and hardware-specific acceleration [41] should be evaluated to confirm real-time feasibility under automotive power and latency constraints.

**Potential ocular artifact confound.** Because SEED-VIG labels are derived from PERCLOS and the preprocessing pipeline does not include ICA-based or regression-based ocular artifact removal, a portion of the signal content in frontal channels may reflect eye-movement artifacts correlated with the labels. The frontal channel ablation experiment (Section 3.4.3) showed that removing the two frontopolar channels (FP1, FP2) reduced LOSO accuracy by only 2.42 percentage points, and the frequency band analysis confirmed that theta (4–8 Hz) rather than delta (0.5–4 Hz, the band most overlapping with EOG) provided the strongest discriminative contribution. These results suggest that the model relies predominantly on cortical information, but a definitive separation of cortical and ocular contributions would require dedicated artifact rejection followed by re-evaluation, which is left to future work.

**Modality and calibration.** The model relies solely on EEG; incorporating additional physiological or behavioral signals could improve robustness under complex driving conditions. The fixed classification threshold does not account for individual differences in baseline spectral profiles, and subject-specific calibration may reduce misclassification near the decision boundary.

**Subject-mixed evaluation design.** The subject-mixed experiments reported in Section 3.2 and Section 3.3 were conducted on class-balanced subsets (400 fatigued + 400 alert samples) rather than on the full eligible data. While the re-sampling stability experiment (Table 7) demonstrates that the reported accuracies are robust to the specific random subset selection, this balanced subsampling design should nonetheless be acknowledged as a limitation of the subject-mixed evaluation. The LOSO protocol (Section 3.5), which uses all eligible data from each held-out subject without subsampling, remains the primary and operationally relevant performance indicator; the subject-mixed results should be interpreted as upper-bound estimates rather than as evidence of real-world deployment performance.

**Cross-subject gap.** The LOSO evaluation (Section 3.5) confirmed that DCAMNet retains its advantage over baselines under a subject-independent protocol, but the 11.92 percentage-point drop from the subject-mixed result (97.35% → 85.43%) underscores that cross-subject generalization remains an open challenge. Domain adaptation, transfer learning, and subject-adaptive fine-tuning are promising directions.

**Cross-labeling-scheme evidence.** The MESD evaluation was included to test whether DCAMNet maintains its advantage under a labeling scheme independent of PERCLOS. An 11-fold LOSO evaluation has been added (Table 5), confirming a 3-point advantage over the strongest baseline, but the smaller subject pool (11 vs. 21 in SEED-VIG) and higher label noise from reaction-time annotation limit the strength of this evidence. No published method has reported LOSO results on MESD under the same LRT/GRT criteria, precluding direct cross-method comparison. Future work could strengthen this evidence by evaluating on additional datasets with other annotation paradigms, such as self-reported drowsiness scales or lane-departure-based labels.

**Temporal modeling.** The current architecture treats each epoch independently and does not model how fatigue accumulates across a continuous drive. Incremental or online learning methods could support longer-term state tracking.

## 5. Conclusions

This paper presented DCAMNet, a lightweight CNN that addresses three signal-level challenges inherent in EEG-based driver fatigue sensing: inter-subject spectral variability, multi-domain feature coupling, and labeling-scheme dependence. Three sequential blocks each target a specific property of the EEG sensor signal: an SE-driven dynamic convolution block adapts spectral sensitivity per sample to accommodate inter-subject variability, a spatial convolution block encodes region-specific cortical activation patterns, and a temporal attention block captures non-stationary fatigue dynamics through windowed variance descriptors and group-wise attention.

The operationally relevant evaluation—leave-one-subject-out cross-validation, which simulates deployment on unseen drivers—yielded 85.43% accuracy on SEED-VIG (2.86-point margin over the strongest baseline; paired *t*-test $p=4.6\times 10^{-10}$, Cohen’s d=2.46) and 79±5% on MESD (3-point margin), demonstrating the benefit of adaptive spectral processing across two independent labeling schemes. Subject-mixed accuracy reached 97.47% (SEED-VIG) and 96.52% (MESD) as upper-bound estimates; these subject-mixed results should be interpreted as secondary to the LOSO findings, as they rely on class-balanced subsampling rather than evaluation on the full eligible data. With only 12.3 K parameters and 1.35 ms inference latency on a standard GPU, DCAMNet achieves these results at a computational cost suggestive of compatibility with resource-constrained embedded sensing platforms, although on-device validation remains necessary. Ablation experiments confirmed that all three blocks contribute non-redundantly, with dynamic convolution providing the largest individual gain. Error analysis revealed that missed detections outnumber false alarms under both protocols, suggesting that threshold adjustment toward higher recall may be warranted in safety-critical deployment scenarios.

Cross-subject generalization remains the principal open challenge: despite the improved LOSO performance reported here, the 11.92 percentage-point gap between subject-mixed and LOSO results—together with the substantial fold-wise variability (±6.51%) across the 21 LOSO folds—reflects the inter-subject variability that persists across all evaluated methods, and reliable cross-subject fatigue detection remains an unresolved challenge for the field. Future work will target this gap through domain adaptation and subject-adaptive calibration, extend the model to multimodal sensor inputs, and validate on-device performance on automotive-grade embedded hardware.

## Figures and Tables

**Table 1:** Sample counts per subject in the MESD dataset (11 of 27 subjects retained after applying the LRT/GRT labeling criteria in Equation (3)).

Data File Name	Subject Number	EEG Segments	
		Fatigued	Non-Fatigued
s01_061102n	1	94	94
s05_061101n	2	66	66
s22_090825n	3	75	75
s31_061103n	4	74	74
s35_070322n	5	112	112
s41_080520m	6	83	83
s42_070105n	7	51	51
s43_070205n	8	132	132
s44_070325n	9	157	157
s45_070307n	10	54	54
s53_090918n	11	113	113
Total		1011	1011

**Table 2:** Layer-wise parameter settings of DCAMNet.

Network Layer	Filter Groups	Dimension	Parameters	Output
Input layer	–	–	–	(C,T)
Filter bank	–	–	–	$\left(N_{b},C,T\right)$
Dynamic convolution layer	*K*	$\left(N_{b},1,L\right)$	$K\times N\times (N_{b}\times L+1)$	$\left(N,C,T_{1}\right)$
Spatial convolution layer	*m*	(C,1)	m×N×C	$\left(m,1,T_{1}\right)$
Time sliding-window layer	–	–	–	$\left(m,T_{1}/w\right)$
Temporal attention layer	*h*	$T_{1}/w$	$h\times (T_{1}/w)$	(m/h,h,1)
Expand layer	–	–	–	(m/h×h)
Fully connected layer	–	–	$N_{c}\times \left(m/h\times h\right)$	$N_{c}$

Note: *C* = number of EEG channels; *T* = number of time points; $N_{b}$ = number of band filters; *N* = number of time filters; $T_{1}$ = number of time points after dynamic convolution; *m* = number of spatial filters; *w* = window length; *h* = number of channels in the temporal attention layer; $N_{c}$ = number of output classes; *K* = number of parallel convolution kernels; *L* = kernel length along the time dimension. The parameter count for the dynamic convolution layer is $K\times N\times (N_{b}\times L+1)$, where $N_{b}\times L$ gives the weights per kernel (input bands × kernel length) and the additional 1 accounts for the bias term. SE branch parameters depend on the reduction ratio *r* and are listed separately in Equation (9).

**Table 3:** Fatigue detection results on the MESD dataset.

Metric	Accuracy	Precision	Recall	F1 Score
Values (%)	96.52	95.23	95.87	95.55

**Table 4:** Comparison of classification results on the MESD dataset (mean ± std over five runs). The best result in each column is in **bold** and the second-best is underlined.

Method	Accuracy (%)	Precision (%)	Recall (%)	F1 Score (%)
CNN	91.38±0.82	90.52±0.91	89.76±1.03	90.14±0.88
LSTM	92.15±0.74	91.28±0.83	90.43±0.95	90.85±0.79
Transformer	92.87±0.69	91.95±0.77	91.12±0.88	91.53±0.74
ResNet	93.62±0.61	92.78±0.70	92.05±0.80	92.41±0.67
DBN	$\underline{94.53\pm 0.55}$	$\underline{93.67\pm 0.63}$	$\underline{93.21\pm 0.72}$	$\underline{93.44\pm 0.60}$
DCAMNet (this work)	96.41±0.47	95.10±0.55	95.72±0.64	95.41±0.51

**Table 5:** Leave-one-subject-out cross-validation results on MESD (mean across 11 folds). The best result in each column is in **bold** and the second-best is underlined.

Method	Accuracy (%)
CNN	71±4
LSTM	70±4
Transformer	73±4
ResNet	74±4
DBN	$\underline{76\pm 5}$
DCAMNet (this work)	79±5

**Table 6:** Fatigue detection results on the SEED-VIG dataset.

Metric	Accuracy	Precision	Recall	F1 Score
Values (%)	97.47	96.71	95.28	95.99

**Table 7:** Re-sampling stability experiment. DCAMNet was trained on five independently drawn balanced subsets per dataset. The original result from the main experiment is shown for reference.

Dataset	Re-Sampling Accuracy (%)	Original Accuracy (%)
SEED-VIG	97.29±0.25	97.35±0.42
MESD	96.34±0.31	96.41±0.47

**Table 8:** Module-level ablation results on SEED-VIG (mean ± std over five runs). The best result in each column is in **bold** and the second-best is underlined.

Variant	Accuracy (%)	Precision (%)	Recall (%)	F1 Score (%)
Full DCAMNet	97.35±0.42	96.58±0.51	95.17±0.63	95.87±0.48
(i) Static conv (K=1, no SE)	95.13±0.57	94.26±0.68	93.41±0.74	93.83±0.61
(ii) w/o Spatial block	95.88±0.49	95.02±0.55	93.95±0.70	94.48±0.53
(iii) w/o Temporal attention	$\underline{96.10\pm 0.46}$	$\underline{95.31\pm 0.52}$	$\underline{94.22\pm 0.65}$	$\underline{94.76\pm 0.50}$
(iv) Static conv + FC only	92.65±0.71	91.80±0.83	90.73±0.91	91.26±0.78

**Table 9:** Effect of the number of parallel kernels *K* on SEED-VIG (mean ± std over five runs). The best result in each column is in **bold** and the second-best is underlined.

K	Accuracy (%)	F1 Score (%)
2	95.78±0.53	94.62±0.58
3	96.84±0.45	95.51±0.50
4	97.35±0.42	95.87±0.48
5	$\underline{97.22\pm 0.47}$	$\underline{95.69\pm 0.52}$

**Table 10:** Effect of the SE reduction ratio *r* on SEED-VIG (mean ± std over five runs). The best result in each column is in **bold** and the second-best is underlined.

r	Accuracy (%)	F1 Score (%)
2	$\underline{97.08\pm 0.48}$	$\underline{95.60\pm 0.54}$
4	97.35±0.42	95.87±0.48
8	96.52±0.55	95.11±0.59

**Table 11:** Effect of frontal channel removal on DCAMNet performance (SEED-VIG). All settings other than the channel set are identical to the main experiments.

Channel Set	Channels	Subject-Mixed Acc. (%)	LOSO Acc. (%)
Full	17	97.35±0.42	85.43±6.51
w/o FP1, FP2	15	96.32±0.53	83.01±6.98
No frontal (FP1/2, F3/4/7/8, FZ removed)	10	92.04±0.81	78.36±7.72

**Table 12:** Leave-one-subject-out cross-validation results on SEED-VIG (mean ± std across 21 folds). The best result in each column is in **bold** and the second-best is underlined.

Method	Accuracy (%)	Precision (%)	Recall (%)	F1 Score (%)
CNN	78.36±8.42	77.51±9.13	76.28±9.75	76.88±8.91
LSTM	79.85±8.15	78.97±8.82	77.94±9.37	78.45±8.56
Transformer	80.46±7.93	79.62±8.58	78.71±9.15	79.16±8.31
ResNet	81.24±7.68	80.42±8.35	79.53±8.92	79.97±8.17
DBN	$\underline{82.57\pm 7.23}$	$\underline{81.69\pm 7.86}$	$\underline{80.85\pm 8.41}$	$\underline{81.27\pm 7.65}$
DCAMNet	85.43±6.51	84.62±7.08	83.76±7.63	84.19±6.89

**Table 13:** Aggregate confusion matrix for DCAMNet under the LOSO protocol on SEED-VIG (summed across 21 folds, 2100 total test samples).

	Predicted Fatigued	Predicted Alert
**Actual Fatigued**	890 (TP)	160 (FN)
**Actual Alert**	150 (FP)	900 (TN)

**Table 14:** Classification results on the SEED-VIG dataset (mean ± std over five runs). The best result in each column is in **bold** and the second-best is underlined.

Method	Accuracy (%)	Precision (%)	Recall (%)	F1 Score (%)
CNN	93.52±0.68	94.45±0.73	92.21±0.81	93.32±0.70
LSTM	94.61±0.59	92.74±0.66	90.58±0.78	91.65±0.64
Transformer	94.83±0.62	93.08±0.69	92.53±0.75	92.80±0.65
ResNet	95.11±0.54	$\underline{95.67\pm 0.60}$	93.94±0.71	$\underline{94.80\pm 0.57}$
DBN	$\underline{96.74\pm 0.48}$	94.41±0.56	95.65±0.62	95.02±0.51
DCAMNet (this work)	97.35±0.42	96.58±0.51	95.17±0.63	95.87±0.48

**Table 15:** Published results on the SEED-VIG dataset for reference. Methods differ in evaluation protocol, preprocessing, sample selection, and input representation; figures are not directly comparable and indicate approximate performance levels only. The best accuracy across the listed entries is shown in **bold** and the second-best is underlined; these markings are provided for visual reference only and should not be interpreted as a head-to-head comparison given the differing protocols. None of the listed methods provide open-source code.

Reference	Method	Accuracy (%)	Protocol
[14]	PLI-GAT	85.53	LOSO
[37]	AGL-Net	87.30	Stratified
[38]	AMD-GCN	89.94	LOSO
[39]	MM-IENET	90.16	Stratified
[40]	MATCN-GT	93.67	Stratified
This study	DCAMNet	**97.47**	Stratified
This study	DCAMNet	85.43	LOSO

**Table 16:** Computational cost on the SEED-VIG configuration (17 channels, 800 time points, binary classification). The smallest parameter count is shown in **bold**. Parameters: sum of requires_grad=True tensors. FLOPs: single-sample forward pass. Inference time: mean ± std over 200 forward passes on an NVIDIA RTX 3090 GPU (NVIDIA Corporation, Santa Clara, CA, USA) after 50 warm-up iterations. The “Relative Params” column reports the parameter count of each baseline relative to DCAMNet (i.e., DCAMNet = 1.0× serves as the reference).

Method	Params (K)	FLOPs (M)	Inference (ms)	Relative Params
CNN	54.4	42.8	1.45±0.38	4.4×
LSTM	54.7	85.3	4.86±0.85	4.4×
Transformer	68.2	115.6	2.84±0.52	5.5×
ResNet	31.1	24.2	2.15±0.44	2.5×
DBN	3515.0	7.3	1.12±0.25	285.8×
DCAMNet (this work)	**12.3**	18.6	1.35±0.33	1.0×

Note: DCAMNet receives the nine-band filter-bank tensor (9×17×800) as part of its multi-view spectral processing, whereas all baselines receive the standard single-band EEG input (1×17×800). The richer input representation is an integral component of DCAMNet’s architecture and accounts for part of its FLOPs. DBN’s high parameter count stems from the flattened input (17×800=13,600 dimensions), but its FLOPs are the lowest because the forward pass involves only matrix multiplications.

**Table 17:** Per-subject LOSO accuracy (%) on SEED-VIG across all 21 folds.

**Subject**	1	2	3	4	5	6	7
Accuracy (%)	91.53	87.24	82.16	89.71	84.38	78.45	86.92
**Subject**	8	9	10	11	12	13	14
Accuracy (%)	90.18	83.67	88.54	91.06	79.33	85.71	82.89
**Subject**	15	16	17	18	19	20	21
Accuracy (%)	73.26	86.41	88.07	80.52	87.63	84.19	81.94

**Table 18:** Single-band evaluation on SEED-VIG. In each row only the indicated band was active; all others were zeroed. The final row shows the full model. The best result in each column is in **bold** and the second-best is underlined.

Band	Range (Hz)	Accuracy (%)	F1 Score (%)
δ	0.5–4	89.37	87.74
θ	4–8	92.33	90.62
$\alpha_{1}$	8–10	91.06	89.39
$\alpha_{2}$	10–13	90.36	89.34
$\beta_{1}$	13–20	87.70	84.75
$\beta_{2}$	20–30	85.72	83.66
$\gamma_{1}$	30–36	84.83	80.85
$\gamma_{2}$	36–42	83.48	80.02
Broadband	0.5–45	94.00	92.63
All bands (full model)	0.5–45	**97.47**	**95.99**

## Data Availability

The MESD dataset used in this study is openly available at https://doi.org/10.6084/m9.figshare.6427334.v5 (accessed on 23 January 2025). The SEED-VIG dataset is available from Shanghai Jiao Tong University at https://bcmi.sjtu.edu.cn/home/seed/index.html (accessed on 23 January 2025); access requires application to the data provider. No new data were created in this study.

## Supplementary Materials

The following supporting information can be downloaded at: https://www.mdpi.com/article/10.3390/s26092728/s1.
