# Supplementary material for: SE-Driven Dynamic Convolution for Adaptive EEG-Based Driver Fatigue Detection Across Spectral, Spatial, and Temporal Domains
Source: Sensors (Basel). 2026 Apr 28;26(9):2728. doi: 10.3390/s26092728 (PMC13165855; doi:10.3390/s26092728)
Supplement: Supplementary file 1 [file sensors-26-02728-s001.zip › sensors-4273495-supplementary.pdf]

Supplementary Material

SE-Driven Dynamic Convolution for Adaptive EEG-Based Driver Fatigue Detection Across Spectral, Spatial, and Temporal Domains

Manuscript ID: **sensors-4273495** | Journal: *Sensors* (MDPI) | Tianle Zhou, Jin Cheng (corresponding), Jinbiao Zhang

S1. Purpose of this document

This document provides the full architectural specifications and the hyperparameter search ranges of the five baseline models used in the controlled comparison reported in Section 3.1 of the main paper. All baselines were trained on the same EEG epochs and labels as DCAMNet, under an identical training pipeline (optimizer, learning-rate schedule, early-stopping criterion, and random seeds). The information below complements the summary descriptions given in Section 3.1 and is intended to support reproducibility.

S2. Common training configuration

Unless explicitly noted in the per-model subsections below, all baselines and DCAMNet shared the same optimizer, learning-rate schedule, and training protocol, as summarized in Table S1.

Table S1. Shared training configuration.

Setting	Value
Optimizer	Adam (β_1=0.9, β_2=0.999)
Initial learning rate	1×10^{-3}
Weight decay	1×10^{-4}
Learning-rate schedule	Step decay: halved every 50 epochs
Maximum epochs	300
Batch size	64
Early-stopping criterion	Validation loss, patience = 30 epochs
Validation split	10% of training data (held out from each fold’s training subjects under LOSO)
Dropout (before final FC)	0.5
Loss function	Cross-entropy (binary)
Random seeds (5 runs)	0, 1, 2, 3, 4
Framework	PyTorch 1.12 on Python 3.8

S3. Baseline-specific architectures

Each baseline received the standard single-band preprocessed EEG input of shape (1, C, T), where C is the number of EEG channels (17 for SEED-VIG, 30 for MESD) and T is the number of time samples per epoch (800 for SEED-VIG, 1500 for MESD). The final fully-connected layer projects to N_c = 2 output classes (alert vs. fatigued).

S3.1 CNN

A lightweight one-dimensional CNN with two temporal convolutional blocks followed by global average pooling and a fully-connected classifier.

Table S2. CNN architecture.

Layer	Configuration	Output shape
Input	—	(1, C, T)
Conv1D #1	64 filters, kernel 5, stride 1, padding 2	(64, C, T)
BN + ReLU	—	(64, C, T)
Conv1D #2	128 filters, kernel 5, stride 1, padding 2	(128, C, T)
BN + ReLU	—	(128, C, T)
Global Avg Pool	over (C, T) dimensions	(128,)
Dropout	p = 0.5	(128,)
FC (classifier)	128 → 2	(2,)

S3.2 LSTM

A two-layer bidirectional LSTM operating on the time dimension after flattening channels into the feature dimension.

Table S3. LSTM architecture.

Layer	Configuration	Output shape
Input	treated as sequence of length T with C features	(T, C)
BiLSTM #1	hidden = 64 per direction (128 total), dropout 0.5	(T, 128)
BiLSTM #2	hidden = 64 per direction (128 total), dropout 0.5	(T, 128)
Final hidden state	concatenate forward/backward last state	(128,)
Dropout	p = 0.5	(128,)
FC (classifier)	128 → 2	(2,)

S3.3 Transformer

A compact Transformer encoder with a prepended classification (CLS) token. The input is linearly projected to the embedding dimension before being processed by two encoder layers.

Table S4. Transformer architecture.

Layer	Configuration	Output shape
Input projection	linear, C → d_{model} = 64	(T, 64)
Positional encoding	learned, length T	(T, 64)
Prepend CLS token	learnable, d_{model} = 64	(T+1, 64)
Encoder layer #1	4 heads, d_{ff} = 256, dropout 0.1, GELU	(T+1, 64)
Encoder layer #2	4 heads, d_{ff} = 256, dropout 0.1, GELU	(T+1, 64)
Take CLS token	position 0	(64,)
Dropout	p = 0.5	(64,)
FC (classifier)	64 → 2	(2,)

S3.4 ResNet (1D)

A one-dimensional adaptation of ResNet-18. Each stage consists of two basic residual blocks with 3×1 convolutions, batch normalization, and ReLU activations. Down-sampling is performed at the first block

of each stage via stride-2 convolutions in the main path and stride-2 1×1 convolutions in the shortcut path.

Table S5. ResNet-1D architecture.

Stage	Configuration	Output channels
Stem	Conv1D (kernel 7, stride 2) → BN → ReLU → MaxPool (kernel 3, stride 2)	16
Stage 1	2 basic blocks, stride 1	16
Stage 2	2 basic blocks, first stride 2	32
Stage 3	2 basic blocks, first stride 2	64
Stage 4	2 basic blocks, first stride 2	128
Global Avg Pool	—	128
Dropout	p = 0.5	128
FC (classifier)	128 → 2	2

S3.5 DBN

A three-layer deep belief network stacked from restricted Boltzmann machines (RBMs). Each RBM was pretrained layer-wise with contrastive divergence (CD-1) for 30 unsupervised epochs; the resulting weights were then used to initialize a feed-forward network fine-tuned with backpropagation under the shared training configuration in Table S1. Because the DBN operates on a flattened input vector, its first layer dominates the parameter count (see Table 16 of the main paper).

Table S6. DBN architecture.

Layer	Configuration	Output shape
Input (flattened)	C × T dimensions (e.g., 17×800 = 13,600 on SEED-VIG)	(C×T,)
RBM #1 / FC #1	(C×T) → 256, sigmoid	(256,)
RBM #2 / FC #2	256 → 128, sigmoid	(128,)
RBM #3 / FC #3	128 → 64, sigmoid	(64,)
Dropout	p = 0.5 (fine-tuning only)	(64,)
FC (classifier)	64 → 2	(2,)

S4. Hyperparameter search space

For each model, the grid below defines the search space that was explored on the validation split (10% of the training data, see Table S1) during preliminary experiments. The value selected for the final reported runs is shown in **bold**. Once selected, the same value was reused across all five random seeds and both evaluation protocols (subject-mixed and LOSO). Values outside the grid were not considered.

Table S7. Hyperparameter search space (selected values in bold).

Model	Hyperparameter	Search grid
All models	Learning rate	1×10^{-4}, 5×10^{-4}, $\mathbf{1 \times 10^{-3}}$, 5×10^{-3}
	Batch size	32, **64**, 128
	Dropout rate	0.3, **0.5**, 0.7
	Weight decay	0, $\mathbf{1 \times 10^{-4}}$, 1×10^{-3}
CNN	Number of conv layers	**2**, 3
	Filters per layer	(32, 64), **(64, 128)**, (128, 256)
	Kernel size	3, **5**, 7
LSTM	Hidden units per direction	32, **64**, 128
	Number of layers	1, **2**, 3
	Bidirectional	**yes**, no
Transformer	Embedding dimension d_{model}	32, **64**, 128
	Number of encoder layers	1, **2**, 4
	Attention heads	2, **4**, 8
	Feed-forward dimension d_{ff}	128, **256**, 512
ResNet	Depth	10, **18**, 34 (1D adaptations)
	Base channels	8, **16**, 32
	Kernel size	3, **7** (stem) / **3** (blocks)
DBN	Hidden layer sizes	(128, 64, 32), **(256, 128, 64)**, (512, 256, 128)
	CD-k pretraining (k value)	**1**, 3, 5
	Pretraining epochs per layer	10, **30**, 50

Note: For fairness across paradigms, the optimizer, learning-rate schedule, early-stopping criterion, and random seeds were not treated as tunable degrees of freedom; they were fixed to the values in Table S1 for every baseline and for DCAMNet. This ensures that differences in reported accuracy reflect architectural differences rather than disparate training budgets.

S5. Hyperparameter selection protocol

For the subject-mixed experiments (Sections 3.2 and 3.3 of the main paper), the 800 balanced samples per dataset were split 7:3 (MESD) or 8:2 (SEED-VIG) by stratified random sampling. Within each training split, 10% of the samples were further held out as an internal validation set (with stratify=y) for hyperparameter selection and early stopping; the test split was never used during model selection.

For the LOSO experiments (Section 3.5), the 10% validation subset used for early stopping and hyperparameter selection was drawn exclusively from the N–1 training subjects in each fold; the held-out subject's data served only as the test set and was never used for validation or model selection. Once a hyperparameter configuration had been chosen on the validation data, the same configuration was applied across all five random seeds (0, 1, 2, 3, 4) to produce the reported mean ± std figures.

End of Supplementary Material.
